# Reviewed article: Silva MT, Amado DM, Rocha PRS, Barreto JO. Integrative and complementary healthcare practices for hypertension: a summary of recommended clinical guidelines. Epidemiol Serv Saude. 2025:34;e20240844

**DOI:** 10.1590/S2237-96222025v34e20240844.b

**Published:** 2025-08-04

**Authors:** Cintia de Freitas Oliveira

**Affiliations:** Instituto de Saúde

**Table:** 

Rating	Excellent	Good	Average	Below average	Poor
Interest	✓				
Quality		✓			
Originality	✓				
Overall	✓				

**Table:** 

Questionnaire	Yes	No	Not applicable
Does the manuscript contain new and significant information to justify publication?	✓		
Does the Abstract (Summary) clearly and accurately describe the content of the article?	✓		
Is the problem significant and concisely stated?	✓		
Are the methods described comprehensively?		✓	
Are the interpretations and conclusions justified by the results?	✓		
Is adequate reference made to other work in the field?	✓		

## Manuscript Structure

Length of article is: Adequate

Number of tables is: Adequate

Number of figures is: Adequate

Please state any conflict(s) of interest that you have in relation to the review of this paper (state “none” if this is not applicable): None

## Comments

O artigo aborda duas temáticas de grande importância para o SUS: o controle da hipertensão arterial sistêmica e a utilização das PICS no manejo da doença e na promoção da qualidade de vida dos pacientes. O artigo está bem estruturado, com clareza na escrita e boa organização das ideias. O método segue as etapas recomendadas para a elaboração de respostas rápidas e os resultados respondem adequadamente à pergunta de pesquisa. Destaco como pontos positivos a utilização da ferramenta AGREE II para avaliar os documentos incluídos e a decisão de incorporar estudos que adotaram o sistema GRADE. Além disso, ter acesso aos resultados dessas avaliações é um aspecto relevante dentro dos processos de tomada de decisão informados por evidências. Portanto, sugiro apenas ajustes pontuais para aprimorar o relato da revisão.

Métodos

1. Os autores tiveram o cuidado de explicar o que é uma revisão rápida, trazendo uma referência importante na área para embasar a conceituação apresentada. No entanto, na linha 35 (página 2), é mencionado que o uso de simplificações metodológicas (atalhos) não compromete a qualidade e a confiabilidade dos resultados. Contudo, é importante ressaltar que o uso de atalhos pode introduzir vieses ao processo de revisão. Apesar de compreender a intenção dos autores em destacar que uma resposta rápida, quando bem conduzida, pode manter boa qualidade e confiabilidade, sugiro que o trecho seja reformulado para evitar possíveis interpretações equivocadas;

2. Ainda em relação aos atalhos metodológicos, sugiro que, no início da seção de métodos, seja destacado quais atalhos foram utilizados na revisão. Da mesma forma, caso os autores considerem que esses atalhos possam ter impactado os resultados, recomendo que essas reflexões sejam incorporadas à discussão, analisando as possíveis implicações de sua utilização, e/ou os cuidados tomados para minimizá-los;

3. Na linha 37 (página 2), trocaria o termo “incluem” por “podem incluir”, considerando que esses são exemplos de atalhos que podem ser adotados em uma resposta rápida, mas não são os únicos, nem precisam ser usados ao mesmo tempo. Caso tenham considerado o item anterior relevante (ponto 2), aqui poderiam ser inseridos os atalhos adotados no presente estudo;

4. Seria interessante também explicar, rapidamente, o porquê da adoção da metodologia das respostas rápidas e não de uma revisão tradicional;

5. Nos critérios de elegibilidade, considero importante caracterizar melhor a população e as intervenções consideradas. No protocolo essas questões estão bem descritas, avaliar, por favor, a possibilidade de acrescentar no texto;

6. Nas estratégias de busca, senti falta do número de registros identificados em cada uma das bases/plataformas de busca. Apenas encontrei o número total de registros identificados no conjunto das três fontes de informação.

7. No item “Avaliação do risco de viés dos estudos”, informar se a avaliação foi feita por um único revisor ou em duplicidade.

Resultados

8. Na Linha 29 (página 4), sugiro manter a frase como “A Tabela 2 caracteriza as Diretrizes Clínicas incluídas”, a fim de evitar repetições na descrição dos resultados que é apresentada ao longo do parágrafo

9. Na linha 33 (página 4), ao descrever a população, os autores informam que os estudos incluíram adultos hipertensos e pacientes com risco cardiovascular elevado. Essas pessoas com risco cardiovascular elevado também eram hipertensas? Devido aos critérios de inclusão imagino que sim, mas avaliem reescrever esse trecho pois fica parecendo, ao menos na minha leitura, que esse grupo não tinha hipertensão, o que seria um critério de exclusão da Diretriz;

10. Ainda em relação à caracterização da população, caso as Diretrizes tenham especificado o tipo de hipertensão, poderiam detalhar essa informação aqui, ou informar que o dado não estava disponível, uma vez que era um dado de interesse da revisão.

